# Proapoptotic BIM Impacts B Lymphoid Homeostasis by Limiting the Survival of Mature B Cells in a Cell-Autonomous Manner

**DOI:** 10.3389/fimmu.2018.00592

**Published:** 2018-03-22

**Authors:** Rui Liu, Ashleigh King, Philippe Bouillet, David M. Tarlinton, Andreas Strasser, Jörg Heierhorst

**Affiliations:** ^1^Molecular Genetics Unit, St. Vincent’s Institute of Medical Research, Fitzroy, VIC, Australia; ^2^Department of Medicine, St.Vincent’s Health, The University of Melbourne, Fitzroy, VIC, Australia; ^3^Molecular Genetics of Cancer Division, Walter and Eliza Hall Institute of Medical Research, Parkville, VIC, Australia; ^4^Department of Medical Biology, The University of Melbourne, Parkville, VIC, Australia; ^5^Department of Immunology and Pathology, Monash University, Melbourne, VIC, Australia

**Keywords:** apoptosis, cell death, BCL-2 family, BIM, BH3-only protein, B lymphocytes, B cell lymphoma

## Abstract

The proapoptotic BH3-only protein BIM (*Bcl2l11*) plays key roles in the maintenance of multiple hematopoietic cell types. In mice, germline knockout or conditional pan-hematopoietic deletion of *Bim* results in marked splenomegaly and significantly increased numbers of B cells. However, it has remained unclear whether these abnormalities reflect the loss of cell-intrinsic functions of BIM within the B lymphoid lineage and, if so, which stages in the lifecycle of B cells are most impacted by the loss of BIM. Here, we show that B lymphoid-specific conditional deletion of *Bim* during early development (i.e., in pro-B cells using *Mb1-Cre*) or during the final differentiation steps (i.e., in transitional B cells using *Cd23-Cre*) led to a similar >2-fold expansion of the mature follicular B cell pool. Notably, while the expansion of mature B cells was quantitatively similar in conditional and germline *Bim*-deficient mice, the splenomegaly was significantly attenuated after B lymphoid-specific compared to global *Bim* deletion. *In vitro*, conditional loss of *Bim* substantially increased the survival of mature B cells that were refractory to activation by lipopolysaccharide. Finally, we also found that conditional deletion of just one *Bim* allele by *Mb1-Cre* dramatically accelerated the development of *Myc*-driven B cell lymphoma, in a manner that was comparable to the effect of germline *Bim* heterozygosity. These data indicate that, under physiological conditions, BIM regulates B cell homeostasis predominantly by limiting the life span of non-activated mature B cells, and that it can have additional effects on developing B cells under pathological conditions.

## Introduction

Members of the BCL-2 family of proteins play crucial roles in the regulation of the mitochondrial apoptotic cell death pathway (1). The BH3-only protein BIM, a proapoptotic member of this family, is particularly critical for regulating cell death/survival in the hematopoietic system. Germline knockout (KO) of *Bim* causes an increase in the number of cells in several hematopoietic subsets, including B cells, T cells, monocytes, and granulocytes, with a marked splenomegaly (2). In these mice, mature B cells are approximately doubled in number compared to wild-type (WT) controls. Upon *in vivo* antigen stimulation, B cells can differentiate into antibody-secreting plasma cells, which are also greatly increased in number in *Bim* KO mice (2). This increase in plasma cells combined with defects in negative selection of autoreactive B cells (3) is thought to lead to the development of a severe auto-antibody-driven systemic lupus erythematosus-like autoimmune pathology with immune-complex glomerulonephritis on a mixed 129SV/JxC57BL/6 genetic background (2). These symptoms of autoimmunity, however, are significantly moderated on a C57BL/6 background (4). *Bim* KO mice on an inbred C57BL/6 background show an abnormally increased proportion of low-affinity B cell receptor (BCR)/surface-IgM expressing B cells in the germinal center, and they accumulate low-affinity memory B cells (5). Both of these cell populations would normally be eliminated by apoptotic cell death during selection for B cells with improved affinity for antigen arising from somatic mutation of their *IgV* genes (5). Conversely, loss of BIM specifically increases the survival of autoreactive immature B cells in the bone marrow, which demonstrated that BIM plays a key role in apoptosis activation by autoreactive BCRs during this developmental stage (3).

BIM expression levels increase progressively during B cell development (pre-pro-B < pro-B/pre-B < immature B < mature B) (6, 7), which may explain why loss of *Bim* has such profound effects on immature and mature B cell populations. However, loss of *Bim* can also increase cell numbers at earlier stages of B cell development under pathological conditions, for example, by supporting the survival (but not proliferation and differentiation) of developing B cells in the absence of IL-7 or the IL-7 receptor *in vivo* and *in vitro* (6, 8). In addition to BIM, other BH3-only proteins such as BMF and PUMA are expressed in B lymphoid cells, and their loss can also lead to increased B cell numbers (7) or synergistically increase B cell numbers in combination with the *Bim* KO (9), highlighting functional redundancies among the proapoptotic proteins.

The B lymphoid expansion resulting from the germline *Bim* KO is transplantable and affects both the follicular and marginal zone compartment (8). In addition, a floxed *Bim* allele has recently been generated, and its conditional deletion throughout the hematopoietic system using *Vav-Cre* recapitulates key features of the germline *Bim* KO phenotype, including increased white blood cell numbers and splenomegaly (10). Collectively, these findings indicate that the B cell-related features of the *Bim* KO phenotype emanate from an impact that is intrinsic to the hematopoietic cell lineage. However, whether these effects on B cell homeostasis are solely due to the loss of a function of BIM specifically within the B lymphoid cell lineage, or whether they may be in part due to an indirect, reactive consequence of losing BIM-dependent apoptosis in another hematopoietic cell type remains unresolved. In addition, if the alterations observed are due to the loss of B cell-intrinsic functions of BIM, it remains to be resolved to what extent they are caused by increased B cell production during their development in the bone marrow, or prolonged survival of mature B cells in the periphery. The relevance of these issues has recently been highlighted by the finding that conditional deletion of *Bim* in myeloid cells (using *LysM-Cre*) can in fact lead to increased splenic B cell numbers and immune-complex glomerulonephritis similar to that observed in germline *Bim* KO mice (11). Thus, to investigate whether BIM regulates B cell homeostasis in a cell-intrinsic manner and to resolve the stage(s) of B cell development at which BIM may exert its most critical functions, we have here employed two different B lymphoid-specific CRE recombinase mouse strains for the conditional deletion of *Bim*: *Mb1-Cre* for deletion during the early developmental pro-B cell stage in the bone marrow (12), and *Cd23-Cre* for deletion at the nearly fully matured transitional B cell stages in peripheral lymphoid tissues (13).

## Materials and Methods

### Mice

Animal experiments were performed according to the Australian Code for the Care and Use of Animals for Scientific Purposes, 8th Edition (2013), and approved by the St. Vincent’s Hospital Melbourne Animal Ethics Committee, approval numbers 019/13 and 002/17.

*Mb1-Cre* (12), *Cd23-Cre* (13), *Eμ-Myc* (14), and *Bim-floxed* (10) mice had been generated, or backcrossed for at least 10 generations, on a C57BL6 background, and were housed in specific pathogen-free micro-isolators. All mice for bone marrow and splenic cell analyses were used at 8 weeks ± 3 days of age, and not selected on gender. Ethical endpoints for tumor-prone mice in survival analyses were determined by trained animal technicians who were blinded to the genotypes of individual mice.

### Immunoblots and Genotyping

Western blotting was performed as described (15) using antibodies against BIM (Cell Signaling Technology, C34C5) and β-actin (EMD Millipore/Merck, MAB1501) as a loading control. DNA from toe biopsies was genotyped using primers 5′-AAGAATCTAGGTTGACTCTAG-3′ and 5′-AACCAACTGTACCTTGGCTATA-3′ resulting in PCR products of ~1 kbp for the *Bim* floxed, ~0.8 kbp for the WT, and ~0.3 kbp for the *Bim*-deleted alleles. In addition, biopsies were genotyped with *Bim* KO primers (5′-AAGAATCTAGGTTGACTCTAG-3′, 5′-CATTGCACTGAGATAGTGGTTGA-3′, and 5′-CCCGTTGCACCACAGATGAA-3′; WT ~0.5 kbp, KO ~0.6 kbp) to exclude mice containing germline *Bim* null alleles as a result of ectopic CRE recombinase activity.

### Cell Preparations

Hind limbs were dissected and skin, muscle, and soft tissues were removed carefully. Both ends of the femur and tibia were cut and the bone marrow was flushed out with 1 mL of ice-cold MACS buffer (1× PBS, pH 7.2, 0.5% BSA, and 2 mM EDTA) using a 21 G needle. Bone marrow cell suspensions from femur and tibia were combined and mixed thoroughly.

Freshly dissected mouse spleens were weighed and gently homogenized in 2 mL cold MACS buffer on ice. Cell suspensions were passed sequentially through 70 and 40-µm cell strainers to remove debris, followed by washes of tubes and strainers with 4 mL MACS buffer to recover remaining cells.

B-1a cells were isolated by peritoneal lavage using 26 and 23 G needles with PBS containing 2% fetal bovine serum (FBS).

Total bone marrow, spleen, and peritoneal cavity leukocyte counts were determined using an automated KX-21N cell counter (Sysmex). Bone marrow cellularities indicated in the figures include tibias and femurs of two legs.

### Flow Cytometry

The following reagents were used for cell staining: B220-APC (eBioscience™, 17-0452-83), B220-FITC (Biolegend, 103206), BP1-PE (BD, 553735), CD11b-pacific blue (eBioscience™, 48-0112-82), CD8-APC (eBioscience™, 17-0081-82), CD4-PE(eBioscience™, 12-0041-83), CD5-FITC (BD, 553021), CD11b-APC-Cy7 (BD, 557657), CD19-APC eFluor780 (eBioscience™, 47-0193-82), CD19-PerCP-Cy5.5 (eBioscience™, 45-0193-82), CD21-PE (Biolegend, 123410), CD23-biotin (BD, 553137), CD24-FITC (BD, 561777), CD43-biotin (BD, 553269), CD43-PE (BD, 553271), GR1-biotin (eBioscience, 13-5931-85), IgD-eFluor450 (eBioscience™, 48-5993-82), IgM-PE-Cy7 (BD, 552867), Brilliant Violet 605™ Streptavidin (Biolegend, 405229), and propidium iodide (Sigma, P4864-10ML). Our gating strategies for staining of bone marrow B lymphoid fractions according to Hardy et al. (16), total and mature splenic B cell numbers, and peritoneal cavity B-1a cell analyses were recently described (15, 17). Hardy fractions were gated as follows: A, B220^+^ CD43^+^ CD24^low^ BP1^−^; B, B220^+^ CD43^+^ CD24^high^ BP1^−^; C, B220^+^ CD43^+^ CD24^low^ BP1^+^; C’, B220^+^ CD43^+^ CD24^high^ BP1^+^; D, B220^+^ CD43^−^ IgM^−^ IgD^−^; E (immature B cells), B220^+^, CD43-, IgM^+^, IgD^−^; F (recirculating B cells), B220^+^, CD43^−^, IgD^+^. T1 transitional B cells were gated as B220^+^, IgM^high^, IgD^low^; T2 transitional B cells as B220^+^, IgM^high^, IgD^high^; and mature B cells as B220^+^, IgM^low^, IgD^high^. Hardy fractions A–D correspond to the Basel classification (18–20) as follows: Hardy A is equivalent to pre-pro-B cells (B220^+^ CD43^+^ CD19^−^); Hardy B/C represent pro-B/pre-B I cells (B220^+^ CD43^+^ CD19^+^ c-kit^+^ CD25^−^ SL^+^); Hardy C’ corresponds to large pre-B II cells (B220^+^ CD43^−^ CD19^+^ c-kit^−^ CD25^+^ SL^+^); and Hardy D corresponds to small pre-B II cells (B220^+^ CD43^−^ CD19^+^ c-kit^−^ CD25^+^ SL^−^). For follicular and marginal zone B cell proportions, splenic cells were first gated on live B220^+^ CD19^+^ lymphocytes, followed by gating on CD21/35 and CD23 (follicular B cells: CD21/35^lo^ CD23^+^; marginal zone B cells: CD21/35^hi^CD23^−^). FACS data were analyzed using FlowJo 10.3 software (Tree Star).

### B Cell Cultures and CellTrace Violet Staining

B cells were isolated from single-cell splenocyte suspensions using B cell isolation kits (Miltenyi Biotec, 130090862) and MACS Separation LS columns (Miltenyi Biotec, 130042401) following the manufacturer’s instructions. 10 million isolated B cells were incubated with CellTrace™ Violet (CTV; Thermo Fisher Scientific, C34557) at 1:1,000 dilution at 37°C in the dark for 20 min, centrifuged at 300 × *g* for 10 min, and the cell pellet was washed once with 10 mL of MACS buffer. CTV-stained B cells were cultured in the B cell culture media (RPMI 1640 supplemented with 5% (v/v) FBS, 50 µM β-mercaptoethanol, 100 U/mL Penicillin G and 100 µg/mL streptomycin sulfate) at a seeding density of 1 million cells/mL. Cells were stimulated with 15 µg/mL lipopolysaccharide (LPS; Jomar Life Research, tlrl-3pelps) or with 1/1,000 recombinant CD40L plus 1/100 conditioned mouse IL4 supernatant for 96 h, and applied to flow cytometry analysis (BD LSRFortessa). Division and proliferation indices were determined using the cell proliferation module of FlowJo 10.3.

### Statistical Analysis

Data from independent experimental replicates were analyzed using GraphPad Prism software. The numbers of independent samples are indicated in the figures and tables. Error bars indicate the mean ± SEM. *p* Values were calculated using the two-tailed unpaired Student’s *t*-test. The Mantel–Cox test was used for survival analyses.

## Results

### Conditional Loss of BIM During Early Stages of B Lymphopoiesis Leads to Increased B Cell Numbers

To analyze cell-intrinsic effects of the loss of BIM on the B cell lineage, we crossed mice containing floxed *Bim* alleles (10) with mice containing the *Mb1-Cre* knock-in allele (12). Within the B lymphoid lineage, *Mb1-Cre* is active from the early pro-B cell stage (12), coinciding with the initiation of *Igh* gene locus rearrangement by V(D)J recombination. However, *Mb1-Cre* can have spurious ectopic activity in the germline (21), especially when transmitted via male breeders. During genotyping, we noticed unusually high frequencies (compared to our experience with other floxed loci, such as *Asciz, Dynll1, tp53*) of ectopic recombination of the floxed *Bim* allele, even in the female germline. Germline heterozygosity for *Bim* can result in some haplo-insufficiency phenotypes, such as reduced life span (2), pronounced acceleration of B cell lymphoma development in *Eμ-Myc* mice (22), and protection from polycystic kidney disease in the absence of *Bcl2* (23). We therefore only used *bona fide Mb1-Cre^ki/+^Bim^fl/fl^* mice for the analyses described here and excluded mice containing inadvertently rearranged germline *Bim*-deleted alleles to avoid any indirect effects from *Bim* heterozygosity in other cell types on the B lymphoid lineage.

**Figure 1:**
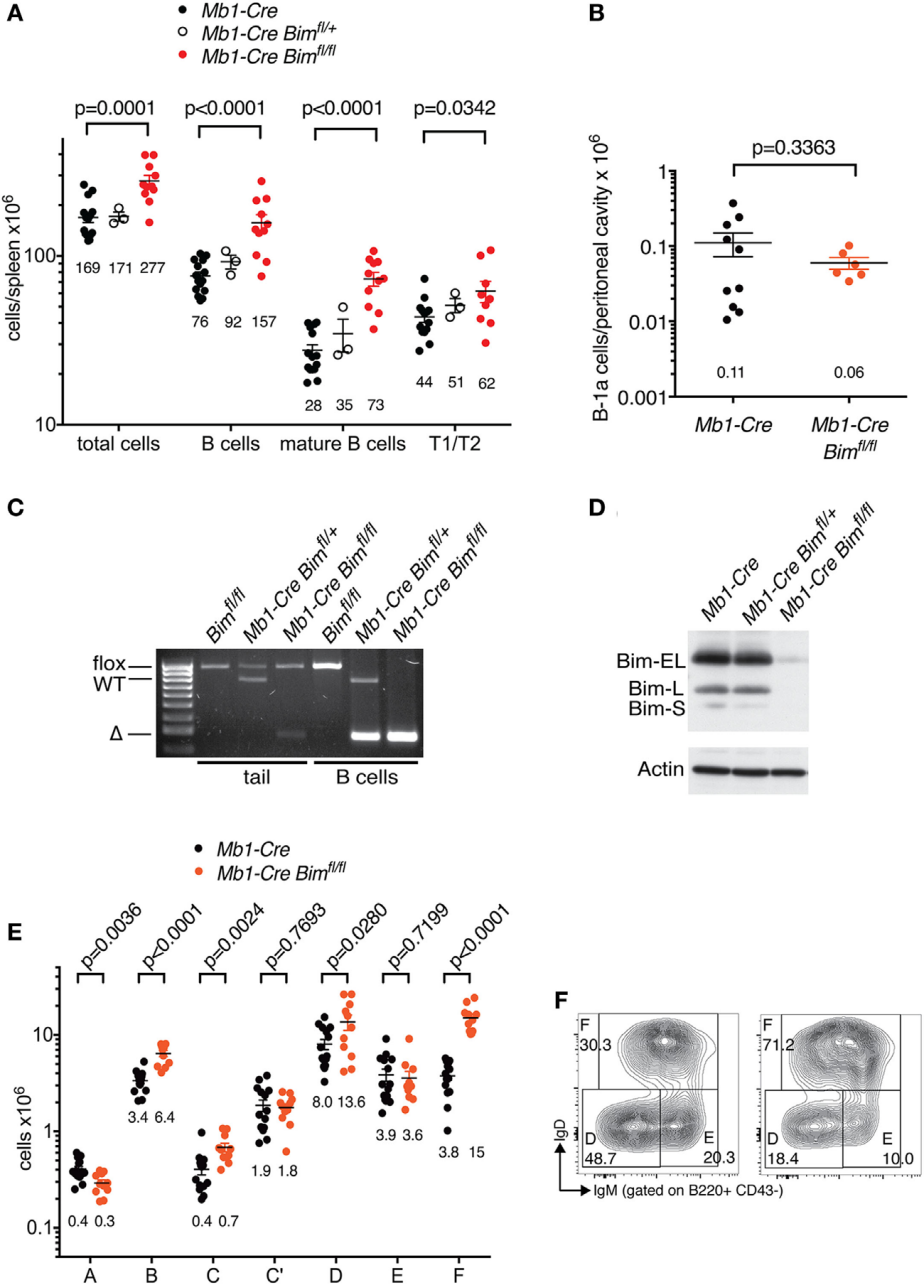
B cell numbers in *Mb1-Cre Bim*-deleted mice. **(A)** Splenic cell numbers. Genotypes of groups are indicated above. Numbers below each group indicate the mean. Numbers for T1 and T2 transitional B cell stages are combined. **(B)** Numbers of B-1a cells in the peritoneal cavity. **(C)** PCR genotyping of genomic DNA isolated from tails or purified B cells of mice of the indicated genotypes. **(D)** Western blot analysis of purified B cells from mice of the indicated genotypes probed with an anti-BIM antibody and actin as a loading control. **(E)** Cell numbers for different developmental stages using the Hardy nomenclature (16). **(F)** Representative FACS plots for bone marrow B cell fractions D (small pre-B), E (immature B cells), and F (recirculating mature B cells).

*Mb1-Cre Bim*-deleted mice contained ~2-fold more total B cells in the spleen than matched *Mb1-Cre Bim^+/+^* control mice (*p* < 0.0001), and this was particularly pronounced among mature B cells, with a much smaller effect on transitional B cells (Figure 1A). In contrast, the numbers of innate-like B-1a cells in the peritoneal cavity [which emanate from distinctive fetal stem cells, as opposed to ongoing hematopoiesis for conventional B cells (24)] were not increased in *Mb1-Cre Bim*-deleted mice (Figure 1B). Efficient B cell-specific *Bim* deletion and loss of all the three BIM protein isoforms [which result from alternative splicing of the *Bim* pre-mRNA (25)] was confirmed by PCR genotyping and immunoblot analyses (Figures 1C,D).

This expansion of the mature B cell pool was also reflected in an ~4-fold increase in recirculating B cells in the bone marrow of *Mb1-Cre Bim*-deleted mice (Hardy fraction F; Figures 1E,F). Conditional *Bim* deletion also led to other significant changes in cellularity during distinct stages of B cell development in the bone marrow. This included a near-doubling of cells in the pro-B cell stage (when *Mb1-Cre* first becomes active; Hardy fraction B) that was carried over into the subsequent early pre-B cell stage (fraction C, when *Igh* V(D)J recombination is completed), and a <2-fold expansion of small pre-B cells (Hardy fraction D, during which *Igl* loci undergo VJ recombination) (Figure 1E). However, *Mb1-Cre Bim*-deleted mice contained normal numbers of cycling pre-B cells (Hardy fraction C’), the stage when cells expressing a successfully rearranged pre-BCR undergo ~5 cycles of clonal expansion. Surprisingly, *Mb1-Cre Bim*-deleted mice also contained normal numbers of immature B cells (Hardy fraction E), the stage when cells expressing self-reactive BCRs are eliminated by a mechanism that involves BIM-dependent apoptosis (3).

Collectively, these data indicate that loss of BIM can affect several stages of B cell development in a lineage-intrinsic manner, and that this is balanced by compensatory changes at other developmental stages.

### Conditional *Bim* Deletion During the Transitional Stages of B Cell Development Leads to Cell-Intrinsic Expansion of the Mature Follicular B Cell Pool

The finding that the numbers of immature B cells (Hardy fraction E), which represent the final stage of B lymphoid development in the bone marrow, were not abnormally increased in *Mb1-Cre Bim*-deleted mice (Figure 1E) suggested that the expansion of the peripheral B cell pools in these mice may be due to an increased life span of mature B cells, rather than increased cell production during development. To test this hypothesis, we intercrossed mice containing floxed *Bim* alleles with mice containing a *Cd23-Cre* transgene. *Cd23-Cre* becomes active in transitional B cells after they have left the bone marrow, and recombination of floxed genes is only complete at the mature B cell stage in peripheral lymphoid organs (13). *Cd23-Cre* did not display ectopic germline activity toward the floxed *Bim* allele.

**Figure 2:**
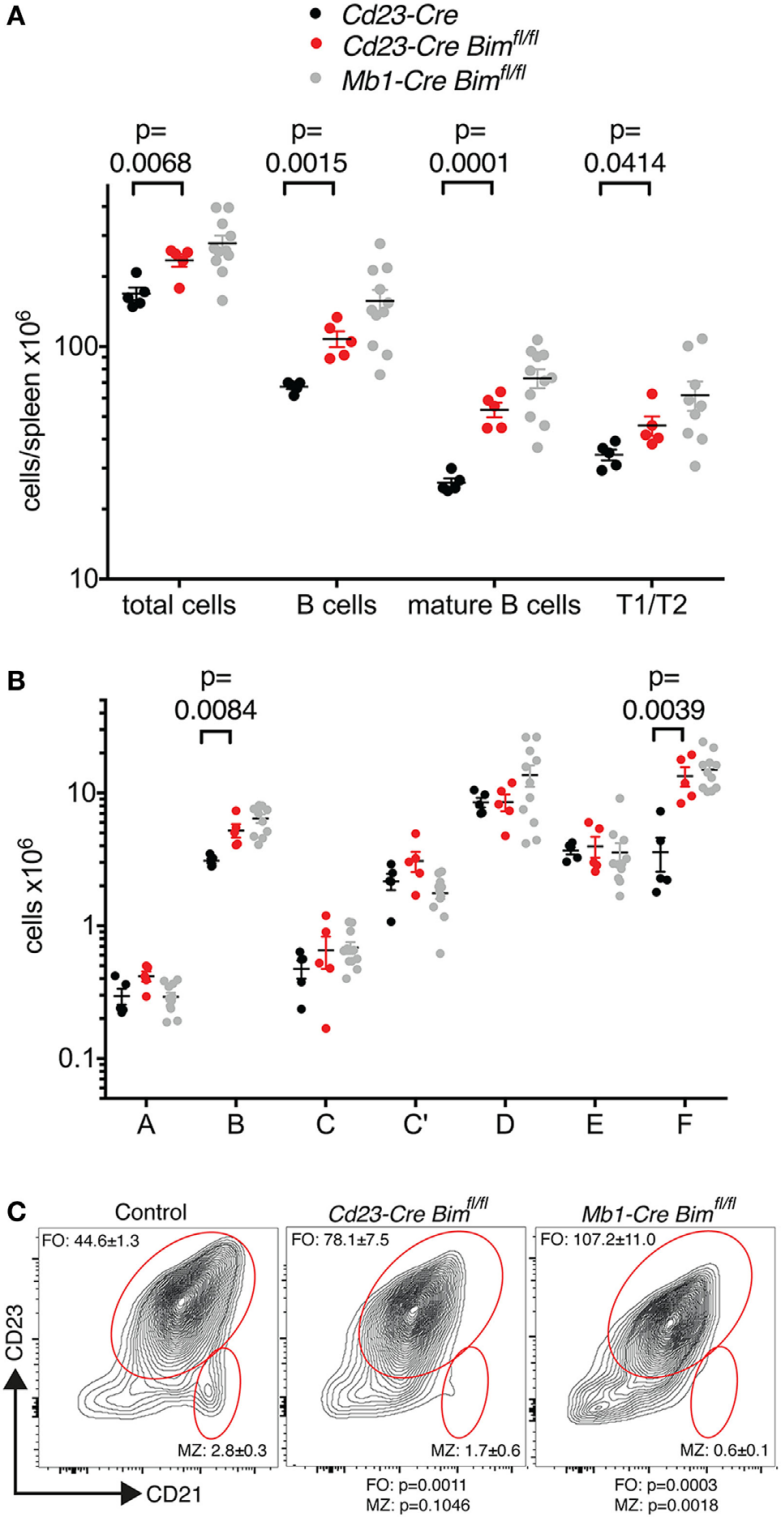
B cell numbers in *Cd23-Cre Bim*-deleted mice. **(A)** Splenic B cell numbers of *Cd23-Cre* control and *Cd23-Cre Bim*-deleted mice. Values for similar analyses of *Mb1-Cre Bim*-deleted mice from Figure 1 are indicated in gray for comparison. Differences between *Cd23-Cre Bim^fl/fl^* and *Mb1-Cre Bim^fl/fl^* mice are not statistically significant. **(B)** Cell numbers for different bone marrow B cell developmental stages. **(C)** Representative FACS plots of B220^+^ IgM^+^-gated spleen cells stained for follicular B cells and marginal zone B cells are indicated. Numbers in the plots indicate the mean ± SE (control, *n* = 5; *Cd23-Cre Bim^fl/fl^, n* = 3; *Mb1-Cre Bim^fl/fl^, n* = 3), and *p* values compared to control mice are indicated below.

Consistent with our hypothesis, *Cd23-Cre*-mediated deletion of *Bim* led to a significant expansion of total and mature B cell numbers in the spleen and in the circulation compared to *Cd23-Cre Bim^+/+^* control mice (Figure 2A; Table 1). The increase in the numbers of mature B cells in *Cd23-Cre Bim*-deleted mice was comparable to the splenic B cell expansion in *Mb1-Cre Bim*-deleted mice (Figure 2A; note that the differences between *Mb1-Cre Bim^fl/fl^* and *Cd23 Bim^fl/fl^* mice were statistically not significant). Further analyses indicated that the expansion of the mature splenic B cell pool was restricted to the CD23-positive follicular B cell compartment, with no increase in the marginal zone B cells (Figure 2C, middle panel). To determine if this specificity for follicular cells was simply due to lower *Cd23-Cre* activity in the marginal zone B cells (which express only very low levels of CD23), we repeated similar analyses using *Mb1-Cre Bim*-deleted mice. Interestingly, loss of *Bim* during early B cell development, before the divergence of follicular and marginal zone cells, again led to a specific expansion of the follicular B cell compartment but not of marginal zone B cells (Figure 2C, right panel). These results indicate that marginal zone B cells are largely indifferent to the loss of *Bim*, which is consistent with the notion that they have a much longer life span than follicular B cells (26).

**Table 1:** Blood cell numbers.

	Control (cells × 10^6^/mL)	*Cd23-Cre Bim^flfl^* (cells × 10^6^/mL)	Significance
**Blood**			
White blood cells	12.53 ± 0.93	20.43 ± 3.05	*p* = 0.0477
B cells	5.270 ± 0.32	12.28 ± 2.51	*p* = 0.0324
CD4^+^CD8^−^ T cells	1.628 ± 0.403	2.336 ± 0.306	
CD8^+^CD4^−^ T cells	1.592 ± 0.292	2.049 ± 0.181	
CD4^+^CD8^+^ T cells	0.069 ± 0.042	0.166 ± 0.147	
Granulocytes	1.868 ± 0.505	1.414 ± 0.537	
Monocytes	1.425 ± 0.227	1.496 ± 0.183	
Red blood cells	8,400 ± 120	8,977 ± 305	
Platelets	950 ± 193	1,157 ± 79	

*n = 4*.

We also enumerated B lymphoid cells in the bone marrow of *Cd23-Cre Bim*-deleted mice. In line with the expansion of the mature B cell pool in the spleen (Figure 2A) and circulation (Table 1), the numbers of recirculating mature B cells (Hardy fraction F) were significantly increased in *Cd23-Cre Bim^fl/fl^* mice compared to control animals, similar to the *Mb1-Cre Bim^fl/fl^* mice. Surprisingly, we also found a significant increase of pro-B cells (Hardy fraction B) in *Cd23-Cre Bim*-deleted mice (Figure 2B). As *Cd23-Cre* is not active at this early developmental stage, this finding indicates that this pro-B cell expansion may be a secondary consequence of the increased mature B cell pool, possibly due to altered cytokine profiles. This indirect expansion of pro-B cells in *Cd23-Cre Bim*-deleted mice, therefore, implies that the quantitatively similar increase of pro-B cells in *Mb1-Cre Bim*-deleted mice may also be due to cell-extrinsic effects. In addition to the effects on the B lymphoid compartment, *Cd23 Bim*-deleted mice also exhibited a modest ~50% increase of splenic CD8^+^ T cells, but other cell types in the circulation and spleen were not significantly changed compared to controls (Tables 1 and 2).

**Table 2:** Splenic cell numbers.

	Control (cells × 10^6^)	*Cd23-Cre Bim^fl/fl^* (cells × 10^**−**6^)	Significance
**Spleen**			
B cells	82.21 ± 3.3.546	117.17 ± 14.28	*p* = 0.0327
CD4^+^CD8^−^ T cells	31.17 ± 2.20	35.93 ± 3.34	
CD8^+^CD4^−^ T cells	16.50 ± 1.06	23.45 ± 2.01	*p* = 0.0141
CD4^+^CD8^+^ T cells	0.655 ± 0.207	1.206 ± 0.205	
Granulocytes	6.885 ± 0.846	4.625 ± 1.075	
Monocytes	6.473 ± 0.42	5.140 ± 0.514	

*Controls are Cd23-Cre^T/+^ Bim^+/+^ mice; n = 4*.

Collectively, these results from the analysis of the *Cd23-Cre*-mediated *Bim* deletion mice indicate that loss of *Bim* within mature B cells is sufficient to increase peripheral B cell numbers in a cell-intrinsic manner, and that this can cause a secondary increase in pro-B cells and CD8^+^ T cells.

### Loss of *Bim* Prevents the Elimination of Activation-Resistant Mature B Cells *In Vitro*

**Figure 3:**
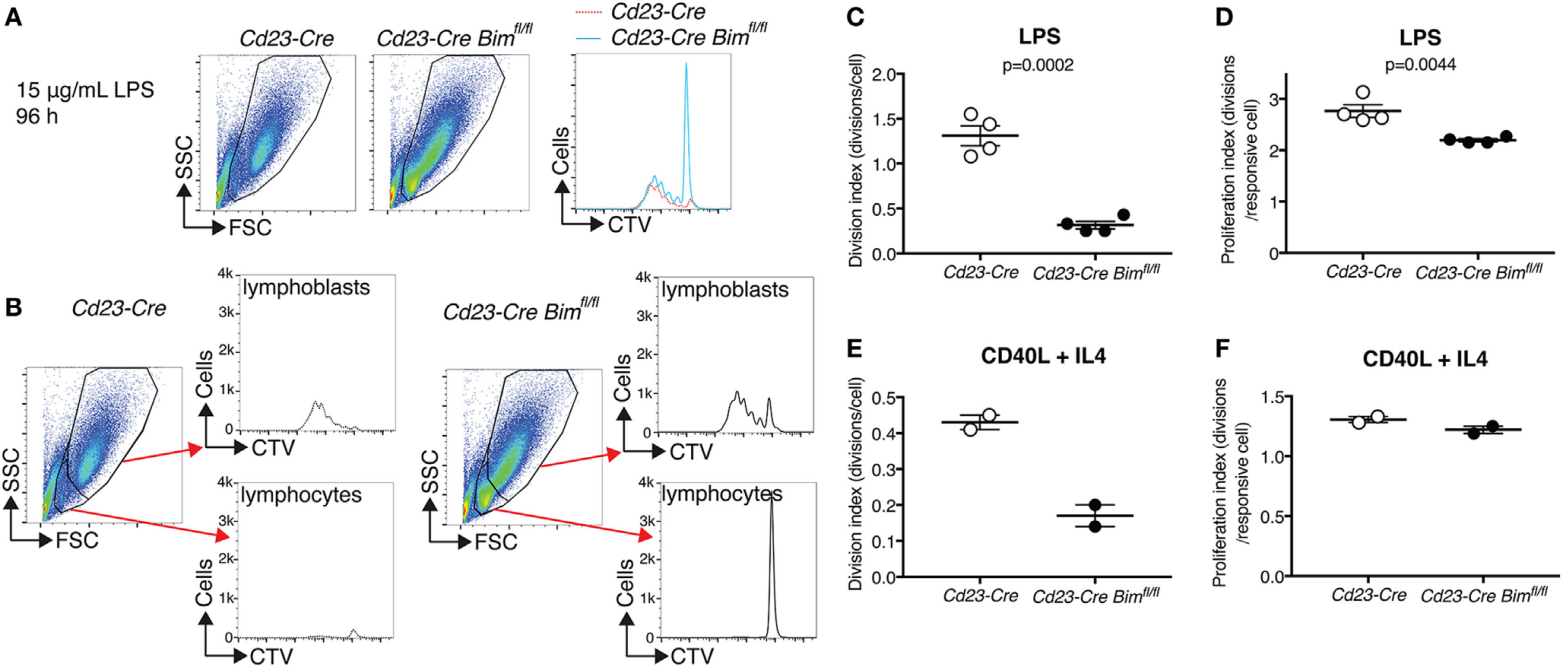
*In vitro* activation of purified B cells. **(A)** Representative FACS plots and CTV-dilution profiles of B cells purified from *Cd23-Cre* control and *Cd23-Cre Bim^fl/f^*^l^ mice after 4 days of treatment with 15 µg/mL lipopolysaccharide (LPS). **(B)** Gating strategies to distinguish small non-responding B lymphocytes from large activated B lymphoblasts, and separated CTV dilution profiles for each fraction. **(C)** Quantification of the cell division index for the entire LPS-treated cultures. **(D)** Quantification of the cell proliferation index (division index excluding cells with undiluted CTV) of LPS-treated cultures. **(E,F)** Cell division and proliferation indices of similar CD40L + IL4-treated B cell cultures.

To assess how loss of *Bim* may lead to increased numbers of mature B cells, we performed *in vitro* B cell activation experiments using LPS and measured proliferation by dilution of CellTrace Violet (CTV). LPS induces a bi-phasic response in mature resting B cells. During the first phase, small B lymphocytes are activated to escape from their G_0_ state by increasing their cell mass, and in the second phase these much larger lymphoblasts enter multiple rounds of cell division (27). It has previously been proposed that LPS activation of B cells leads to the inactivation of BIM as a result of multisite phosphorylation by ERK and subsequent ubiquitin-dependent proteasomal degradation (28). Interestingly, LPS-treated *Cd23-Cre Bim*-deleted B cells produced a similar number of dividing cells and a similar CTV dilution profile compared to *Cd23-Cre* control B cells (Figure 3A). However, whereas the small, non-blasting and undivided lymphocytes were almost completely lost from the cultures of control B cells after 4 days of LPS treatment, a substantial number of these resting cells survived in the cultures of *Cd23-Cre Bim*-deleted B cells (Figure 3B). Analysis of the CTV dilution assays, using the proliferation platform of the FlowJo software package, indicated that relative to the numbers of remaining undivided cells, there was a ~4-fold reduction in the average number of divisions per cell in the cultures of *Bim*-deleted B cells compared to the *Cd23-Cre* control B cells (Division index, Figure 3C). This difference was reduced to ~0.2-fold fewer divisions when only the activated cells that had divided at least once were taken into consideration (Proliferation index, Figure 3D). Qualitatively similar results were also obtained in response to treatment with CD40L (Figures 3E,F). Taken together, these results indicate that loss of BIM primarily increases the survival of activation-resistant resting B lymphocytes that do not exit the G_0_ state, with a relatively minor effect on the survival of actively proliferating B lymphoblasts.

### Heterozygous Conditional *Bim* Deletion in Developing B Cells Accelerates MYC-Driven B Cell Lymphomagenesis

While the above experiments indicate that under normal physiological conditions B cell-intrinsic loss of *Bim* exerts its main effect on the mature B cell pool, there are several instances where germline loss of *Bim* can have profound effects on murine B cell development under pathological conditions (6, 8, 29). For example, in the context of *MYC*-driven B cell lymphomagenesis, it has been shown that the loss of even a single copy of *Bim* leads to dramatically accelerated lymphoma development emanating from the pre-B and immature B cell stages in the bone marrow of *Eμ-Myc* transgenic mice (22). We therefore also compared the effects of heterozygous germline versus heterozygous B lymphoid-intrinsic conditional *Bim* deletion on the time course of lymphoma development in *Eμ-Myc* mice.

**Figure 4:**
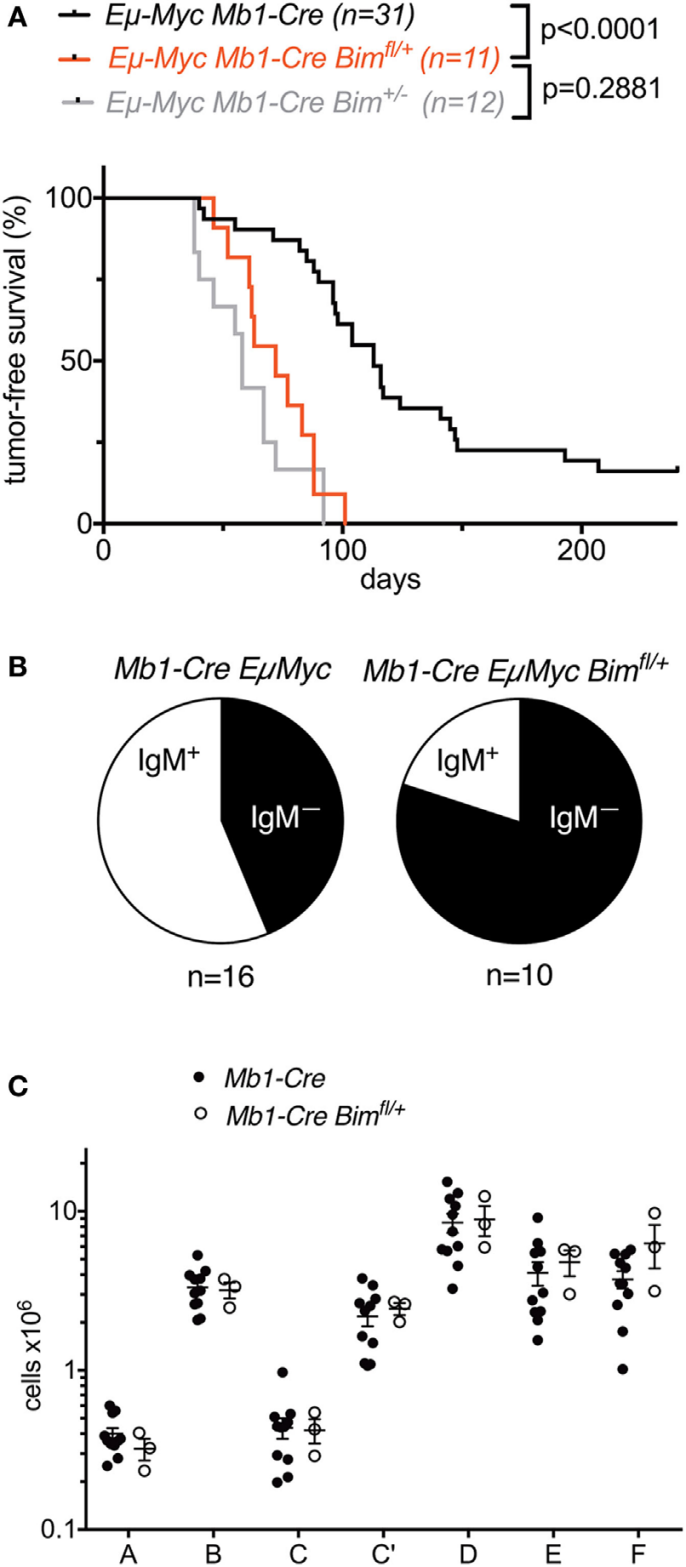
Effect of conditional heterozygous *Bim* deletion on B cell lymphoma development in *Eμ-Myc* transgenic mice. **(A)** Survival analysis of *Eμ-Myc Mb1-Cre Bim^fl/+^* mice compared to *Eμ-Myc Mb1-Cre* control mice and *Eμ-Myc Mb1-Cre Bim^±^* mice. Numbers for *Eμ-Myc Mb1-Cre Bim^±^* mice are from the study by Wong et al. (15). **(B)** Proportions of surface IgM-positive or surface IgM-negative lymphomas in the control and conditional *Bim*-heterozygous cohorts. **(C)** Comparison of bone marrow B cell developmental cell fractions in control and conditional *Bim*-heterozygous groups.

Compared to *Mb1-Cre Eμ-Myc* control mice (that also contained an *Mb1-Cre* knock-in allele; median survival 113 days), conditional *Mb1-Cre Bim^fl/+^Eμ-Myc* mice (median survival 72 days) exhibited significantly accelerated lymphoma development (Figure 4A). The survival curve for these B lymphoid-specific conditional *Bim* heterozygous mice was not significantly different from the survival curve for otherwise isogenic germline *Bim* heterozygous mice [that also contained an *Mb1-Cre* knock-in allele (15)] (Figure 4A). Among the tumors that formed in the *Mb1-Cre Bim*-deleted *Eμ-Myc* mice, there were higher proportions of surface IgM-negative precursor B cell lymphomas compared to the *Mb1-Cre Eμ-Myc* control mice (Figure 4B), although this difference was statistically not significant. It should be noted that heterozygous conditional *Bim* deletion did not affect normal B cell development compared to *Mb1-Cre* control mice (Figure 4C). Overall, these data indicate that the effect of *Bim* heterozygosity on the lymphoma development of *Eμ-Myc* transgenic mice is largely B lymphoid-intrinsic, without quantifiably significant compounding effects of *Bim* deficiency in other tissues, including their developmental niche in the bone marrow.

## Discussion

**Figure 5:**
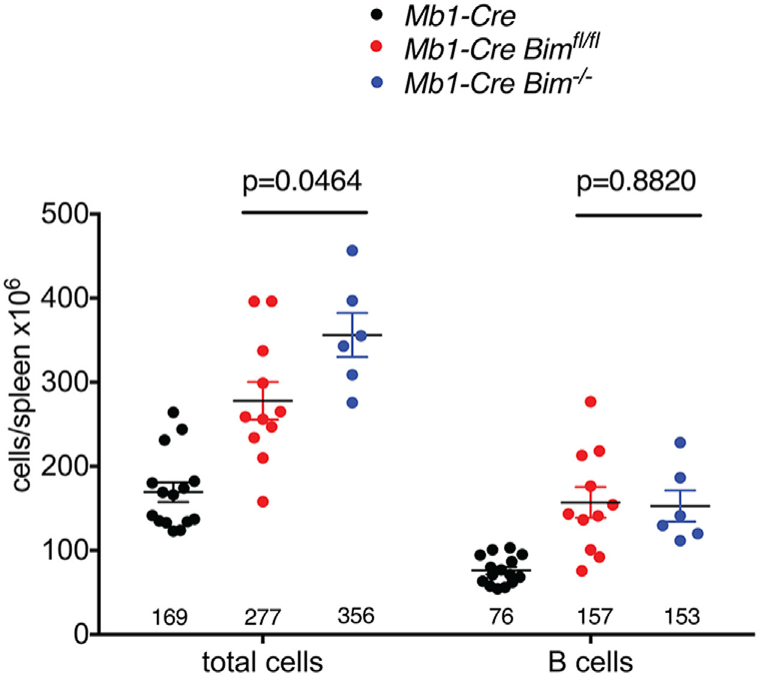
Comparison of splenic cell numbers between conditional and germline *Bim*-null mice. Numbers for *Mb1-Cre* control and conditional *Mb1-Cre Bim^fl/fl^* mice are from Figure 1. Numbers for germline *Mb1-Cre Bim^−/−^* mice are from the study by Jurado et al. (29).

One of the most prominent phenotypes of germline *Bim* KO mice is a profound increase in the number of peripheral B cells, and an associated splenomegaly, but it was unclear to what extent this was due to B lymphoid-specific functions of BIM or influenced by the absence of BIM in other tissues and hematopoietic cell types. Notably, the numbers of splenic B cells in the conditional *Mb1-Cre Bim*-deleted mice reported here are strikingly similar to those of germline *Bim* KO mice (on the same genetic background, at the same age and under the same housing conditions, albeit at different times) that also contained a *Mb1-Cre* knock-in allele (29) (Figure 5). In contrast, the total spleen cell numbers were significantly more increased in the germline *Bim*-null mice compared to the B lymphoid-specific *Bim*-deleted mice (Figure 5). These data therefore indicate that the B cell expansion in the *Bim* KO is largely due to cell-intrinsic changes within the B-lymphoid cell lineage, but the loss of *Bim* in other cell types is also an important contributor to the severity of the splenomegaly.

The pool of mature B cells is constantly regenerated in mice in unison with the elimination of non-reactive or dormant B cells that are not encountering antigens specific to their unique BCRs (30, 31). This raises the question whether loss of BIM affects B cell numbers by regulating the generation of new B cells or by regulating the survival of existing B cells. In addition to having increased numbers of mature B cells, the conditional *Mb1-Cre Bim*-deleted mice also contained more pro-B and pre-B cells than control animals (fractions B, C, and D; Figure 1E). However, this expansion of B lymphoid cells from earlier developmental stages was reversed to normal cellularity at the immature B cell stage (fraction F; Figure 1E), which represents the final stage before developing B cells emigrate from the bone marrow to peripheral lymphoid tissues including the spleen. This may be due to BIM-deleted cells undergoing fewer divisions at the large pre-B cell stage, perhaps due to limited availability of growth factors that drive this cell proliferation, or the elimination of excess cells by BH3-only proteins other than BIM. For example, the *Bmf* KO leads to more pronounced elevation of pre-B cells and immature/transitional B cells in the bone marrow than the *Bim* germline KO (7). Regardless, the observation that *Mb1-Cre Bim*-deleted mice have increased numbers of mature B cells but normal numbers of immature B cells suggests that the expansion of mature B cells caused by the absence of BIM may largely be due to their increased life span rather than increased survival of cells at earlier stages of B lymphopoiesis. This notion is supported by our analysis of conditional *Cd23-Cre Bim* mice, which delete *Bim* only during the final differentiation stages after B lymphoid cells have left the bone marrow, yet exhibit a quantitatively comparable peripheral mature B cell expansion to the *Mb1-Cre Bim* mice.

The conclusion of our *in vivo* analyses, that BIM regulates B cell homeostasis primarily through the stochastic elimination of superfluous mature B cells, is also corroborated by our *in vitro* data, which show that loss of BIM had a much more pronounced effect on the survival of activation-resistant mature B lymphocytes than on the proliferation of activated B lymphoblasts (Figure 3). It should be noted that these findings for *in vitro*-cultured, conditionally *Bim*-deleted cells are consistent with earlier *in vitro* findings for purified B cells from germline *Bim* KO mice (28), where loss of BIM had a much more pronounced effect on apoptotic cell death of untreated resting cells than on the survival of LPS-stimulated cells after 24–48 h in culture. The latter suggests that other proapoptotic BH3-only proteins, possibly PUMA (5), or other cell death mechanisms, such as death receptor-mediated apoptosis (32), may be more important than BIM in the killing of activated B lymphoblasts.

While the considerations above relate to how BIM regulates B cell homeostasis under physiological conditions in otherwise normal mice, it has previously been shown that germline loss of *Bim* can also impact B cell development, for example, in the context of other mutations or under pathological conditions. A prominent example of this is the accelerated development of B cell lymphomas emanating from pre-B cells or immature B cells in *Eμ*-*Myc* transgenic mice (22). Our data indicate that the conditional and germline loss of *Bim* have quantitatively comparable effects in this context. Other examples of the effect of BIM on B cell development include the partial or complete rescue of B cell development defects of IL-7-deficient or IL-7 receptor-deficient mice (6, 8), *dicer*-deficient mice (33), or *Asciz*-deficient mice (29) by germline *Bim* KO. Indeed, we recently found that conditional *Bim* deletion can fully rescue the B cell developmental defects of *Mb1-Cre Dynll1*-deleted mice (17), which are mechanistically and quantitatively similar to those observed in the *Mb1-Cre Asciz*-deleted mice. This indicates that BIM exerts its effects on B cell development under pathological conditions, at least during B lymphomagenesis, also in a cell-intrinsic manner in the B lymphoid lineage.

Finally, while our data indicate that the expansion of mature B cells in *Bim* KO mice is largely driven in a cell-intrinsic manner, it has been reported that substantial B cell expansions can also be triggered by the conditional deletion of *Bim* within only the myeloid cell compartment (11). However, it should be noted that these analyses of *LysM-Cre Bim*-deleted mice were performed in much older mice (8 months versus 8 weeks of age for our mice) and after they had developed a multi-organ autoimmune syndrome. Nevertheless, these findings highlight that it would be worthwhile to monitor the *Cd23-Cre Bim*-deleted mice for longer periods to determine if B cell-specific loss of BIM eventually leads to age-dependent deposition of immune complexes in susceptible tissues, such as the glomeruli in the kidney, and the development of any autoimmune syndrome-like pathologies, although these would be expected to be relatively mild on a C57BL/6 background (see above).

## Ethics Statement

Animal experiments were performed according to the Australian Code for the Care and Use of Animals for Scientific Purposes, 8th Edition (2013), and approved by the St. Vincent’s Hospital Melbourne Animal Ethics Committee, approval numbers 019/13 and 002/17.

## Author Contributions

RL was involved in design, performing and analysis of experiments, and contributed to drafting of the manuscript. AK was involved in design, performing, and analysis of experiments. PB, DT, and AS provided resources and were involved in discussing experiments and editing the manuscript. JH was involved in the conception of the study, the design, analysis, and supervision of experiments, and drafting and editing of the manuscript.

## Conflict of Interest Statement

The authors declare that the research was conducted in the absence of any commercial or financial relationships that could be construed as a potential conflict of interest.
